# CXCR4-directed theranostics in oncology and inflammation

**DOI:** 10.1007/s12149-018-1290-8

**Published:** 2018-08-13

**Authors:** Malte Kircher, Peter Herhaus, Margret Schottelius, Andreas K. Buck, Rudolf A. Werner, Hans-Jürgen Wester, Ulrich Keller, Constantin Lapa

**Affiliations:** 1 Department of Nuclear Medicine, University Hospital Würzburg, Oberdürrbacher Str. 6, 97080 Würzburg, Germany; 2 Internal Medicine III, Hematology and Medical Oncology, Technische Universität München, Munich, Germany; 3 Pharmaceutical Radiochemistry, Technische Universität München, Munich, Germany; 4 Division of Nuclear Medicine and Molecular Imaging, Johns Hopkins University School of Medicine, Baltimore, USA

**Keywords:** Chemokine, Cancer, Theranostics, Pentixafor, Pentixather

## Abstract

Given its prominent role in inflammation and cancer biology, the C-X-C motif chemokine receptor 4 (CXCR4) has gained a lot of attention in the recent years. This review gives a short overview of the physiology and pathology of chemokines and chemokine receptors and then focuses on the current experience of targeting CXCR4, using radiolabeled receptor ligands suitable for positron emission tomography (PET) imaging, in both hematologic and solid malignancy as well as in inflammatory conditions. Additionally, CXCR4-directed endoradiotherapy (ERT) as a new treatment option is discussed.

## Introduction

### Chemokines and chemokine receptors

Chemokines are small, secreted proteins that are defined by their structurally important cystein residues, and therefore, grouped, according to the systemic nomenclature from 2000, into four subfamilies, namely CC, CXC, CX_3_C and XC chemokines [1]. Chemokine receptors are named according to their respective ligand (chemokine) and are divided into two groups—conventional chemokine receptors (cCKRs) and atypical chemokine receptors (ACKRs) [2]. cCKRs belong to the family of G protein-coupled receptors, and therefore, typically signal via the MAPK- and β-arrestin pathway. ACKRs structurally resemble cCKRs but are not coupled to G proteins. Therefore, they are mainly involved in the scavenging and consequently the homeostasis of chemokines [3].

### Role of CXCR4 and its ligand CXCL12 in physiology and pathology

The chemokine receptor CXCR4 is a seven transmembrane G protein-coupled receptor. CXCR4 is widely expressed throughout the human body during embryonic development and adult life, with uniquely high-expression levels in the hematopoietic system. Its cognate ligand, the chemokine CXCL12 (also named stromal cell-derived factor-1α, SDF-1α), is mainly expressed in the bone marrow (BM), lymph nodes, lung, heart, thymus and liver [4]. The canonical CXCR4-CXCL12 axis activates major cellular signaling pathways like RAS-MAPK, PI3K-AKT-mTOR, JAK-STAT and PLC. The β-arrestin pathway displays a negative feedback loop, leading to CXCR4 internalization and its lysosomal degradation [5].

The outstanding role of the CXCR4-CXCL12 pathway within the chemokine network is emphasized by the fact that either a CXCR4 or CXCL12 deletion, by means of gene knockout, results in embryonic lethality in mice. This reflects the importance of the signaling axis during the development of the hematopoietic, nervous and cardio-vascular system [6–8]. Apart from its role in organogenesis, CXCR4-CXCL12 signaling is crucially involved in the homeostasis of the adult hematopoietic system, mainly due to its implication in the retention of hematopoietic stem cells in the BM niche [9]. Beyond, it orchestrates an adequate response of the adoptive and innate immune system.

However, the CXCR4 receptor has also been found to be involved in a variety of diseases. For example, it mediates HIV-1 entry into T cells as a co-receptor, where it was first identified [10]. Furthermore, in rheumatoid arthritis, CXCR4-expressing CD4^+^ memory T cells accumulate in the inflamed synovium due to the locally increased CXCL12 concentration [11]. In the pathogenesis of atherosclerosis, CXCR4 is involved in the chronic inflammation of the arterial wall which is characterized by a chemokine-mediated influx of leukocytes [12]. CXCR4 has also been identified as a key player in vascular remodeling after injury, atherosclerotic plaque destabilization and aneurysm formation [13]. Moreover, chronic inflammation, and thus local infiltration with CXCR4-expressing immune cells, strongly promotes carcinogenesis of esophageal cancer [14]. Aside from its involvement in various inflammation-related processes, CXCR4 dysregulation was also found to significantly contribute to neurodegenerative diseases [15].

### CXCR4-CXCL12 role in cancer

CXCR4 and CXCL12 play a pivotal role in tumor development and metastasis [16, 17]. This has been demonstrated for a variety of cancer entities, including breast [18], prostate [19, 20], lung [21, 22] and colorectal cancer [23], as well as primary brain tumors such as glioblastoma [24]. Overall, the level of CXCR4 and CXCL12 expression is predictive for the metastatic potential of a given tumor type and mediates organ-specific metastasis [25]. In fact, chemokines are at the center of molecular control of metastasis and tumor growth [26]. By activation of various signaling pathways, e.g., RAS-MAPK, PI3K-AKT-mTOR and JAK-STAT, the CXCL12-CXCR4 axis promotes tumor proliferation, inhibits apoptosis of cancerous cells and facilitates metastasis [27]. CXCL12 modulates the tumor microenvironment by autocrine and paracrine secretion. For instance, the attracted stromal cells are stimulated to secrete growth factors that support tumor proliferation and angiogenesis [27–30]. Further, high CXCL12 levels—via the activation of NF-ĸB—suppress the production of TNF-α which subsequently leads to a protection of tumor cells from entering apoptosis [31, 32]. In addition, CXCL12 modulates the immune response to the tumor tissue, e.g., by recruiting dendritic cell populations. Those cells tolerate tumor tissue due to a dysfunction in their tumor-associated antigen-presentation to T cells, thereby promoting immunosuppression within the tumor microenvironment [33, 34]. Hence, the disruption of the CXCL12-CXCR4 axis provides a promising molecular target for future specific cancer therapies.

### Targeting the CXCR4–CXCL12 axis

Given the undisputed clinical relevance of CXCR4 concerning the growth and spreading of a variety of malignancies, a multitude of CXCR4-directed peptidic and non-peptidic antagonists have been developed during the last decade [16, 28]. Amongst them, the bicyclam AMD3100 (Plerixafor/Mozobil™) is the only compound that has been approved by the FDA (in 2008) for the mobilization of stem cells and for the treatment of hematological malignancies and other cancers [35–38]. In preclinical mouse models of various malignancies, CXCR4-directed therapies using either alternative small-molecule CXCR4 antagonists such as AMD3465 [39, 40] or MSX-122 [41], peptidic CXCL12 derivatives (CTCE-9908 [42], BKT-140 [43–45], POL-5551 [46–48]), anti-CXCR4-antibodies [49–52] or CXCL12 inhibitors such as the Spiegelmer Nox-A12 [53], have been shown to consistently lead to prolonged overall survival, primarily by effectively preventing distant organ metastasis [54]. Another potent CXCR4 antagonist, LY2510924 (cyclo[Phe-Tyr-Lys(iPr)-d-Arg-2-Nal-Gly-d-Glu]-Lys(iPr)-NH_2_) [55, 56], exhibited high antitumor activities in solid tumor and breast cancer metastatic models and is currently evaluated in phase II clinical trials. Recently, disulfide-bridged cyclic heptapeptide antagonists with excellent in vivo stability [57, 58] have been shown to efficiently inhibit lung metastasis in a melanoma model [59], and to reduce the metastatic potential of hepatocellular carcinoma and osteosarcoma in a mouse model [60]. A modified analog (R29, Ac-Arg-Ala-[d-Cys-Arg-Phe-His-Pen]–COOH) efficiently reverts the suppressive activity of T regulatory cells in renal cancer [61]. Lastly, overcoming chemoresistance in AML via RNA-interference within the CXCR4–CXCL12 axis was examined in a human AML xenograft model [62].

### Tracer development for diagnosis and therapy

Based on these developments and to meet the clinical need for pre-therapeutic quantification of CXCR4 expression, intense efforts have also been directed towards the development of suitable CXCR4-targeted molecular imaging agents [63, 64]. Amongst the mentioned CXCR4-targeted antagonists, three classes of compounds have been extensively evaluated with respect to their suitability as in vivo CXCR4 imaging agents:

- radiolabeled analogs of the bicyclams AMD3100 [65–67] and AMD3465 [68–70],
- ^18^F- or ^68^Ga-labeled T-140-based peptides for PET imaging as well as corresponding nuclear/fluorescent ligands for optical/SPECT imaging [71–78], and
- radiolabeled, FC-131-based cyclic pentapeptides [79–86].

From all three classes, highly promising candidates with high CXCR4 affinity and excellent CXCR4-targeting properties in vitro and in vivo in preclinical studies have emerged, and single representatives such as [^64^Cu]AMD3100 [65], the T-140 analogue [^68^Ga]NOTA-NFB [74] and the cyclic pentapeptide [^68^Ga]Pentixafor [80, 85] have also been evaluated in patients. Unfortunately, the clearance pattern of the first two compounds, both of which exhibit considerable to very high splenic and liver uptake in mice and humans, challenge their applicability for high contrast clinical imaging of CXCR4 expression. In contrast, the FC-131-derived analogue [^68^Ga]Pentixafor cyclo(D-Tyr^1^-D-[NMe]Orn^2^(AMBS-[^68^Ga]DOTA)-Arg^3^-Nal^4^-Gly^5^) [79, 80, 85, 87, 88] shows high affinity and selectivity for human CXCR4, rapid renal excretion, and very low non-specific background accumulation, allowing sensitive and high-contrast PET imaging of CXCR4-expressing tissues in vivo and thus is the only CXCR4-targeted imaging agent that has found broad clinical applicability so far.

Unfortunately, the pronounced sensitivity of the Pentixafor scaffold towards even minor structural modifications [82], that ultimately lead to strongly decreased CXCR4 affinity, precludes the use of its ^177^Lu- or ^90^Y-labeled version as the corresponding CXCR4-targeted endoradiotherapeutic (ERT) agent within a theranostic concept. Thus, a closely related alternative peptide backbone (cyclo(d-3-iodo-Tyr^1^-d-[NMe]Orn^2^-Arg^3^-Nal^4^-Gly^5^)) was chosen for the realization of a first CXCR4-targeted endoradiotherapeutic agent, namely Pentixather (cyclo(d-3-iodo-Tyr^1^-d-[NMe]Orn^2^(AMBS-DOTA)-Arg^3^-Nal^4^-Gly^5^)) [84]. The efficacy and toxicity of Pentixafor/Pentixather-based CXCR4-targeted theranostic approach was demonstrated in patient-derived (PDX) and cell line-based xenograft mouse models of ALL and AML [89]. Here, [^68^Ga]Pentixafor PET enabled visualization of CXCR4-positive leukemic burden, and CXCR4-directed ERT with [^177^Lu]Pentixather resulted in the efficient reduction of leukemia in leukemia-harboring tissues (spleen, bone marrow). Despite a substantial in vivo cross-fire effect to the leukemia microenvironment, mesenchymal stem cells subjected to ERT were viable and capable of supporting the growth and differentiation of non-targeted normal hematopoietic cells ex vivo [89].

### CXCR4 imaging in oncology

#### Imaging hematologic malignancies

As a likely consequence of its high physiological expression on normal hematopoietic cells, CXCR4 surface levels are also particularly high in several hematologic malignancies, including non-Hodgkin lymphoma (NHL), multiple myeloma (MM), chronic lymphocytic leukemia (CLL) and acute myeloid leukemia (AML), with substantial heterogeneity between diseases and within single entities.

Thus, as a proof-of-concept, the first clinical application of [^68^Ga]Pentixafor for CXCR4-directed PET imaging has been carried out in patients with lymphoproliferative diseases, i.e., NHL and MM [85]. Since then, most experience with [^68^Ga]Pentixafor PET imaging has been gained in patients with MM. In the first disease-specific, proof-of-concept investigation Philipp-Abbrederis et al. showed that [^68^Ga]Pentixafor PET was able to image disease manifestation in 10/14 patients with MM [90]. These results were confirmed in a larger study by Lapa et al. in which CXCR4 overexpression was shown in lesions of 23/34 MM patients upon CXCR4-targeted PET imaging [91]. Importantly, in both studies, CXCR4-directed PET with [^68^Ga]Pentixafor provided additional information concerning lesion numbers in comparison to [^18^F]FDG PET.

Further proof-of-concept studies have shown the clinical applicability of [^68^Ga]Pentixafor in AML and CLL. Herhaus et al. showed that in AML, where the CXCR4-CXCL12 axis is crucially involved in attraction and retention of leukemic cells into the protective BM niche, CXCR4-directed imaging with [^68^Ga]Pentixafor was able to identify patients with CXCR4-positive AML [92]. Another study with [^68^Ga]Pentixafor revealed that BM involvement in CLL patients is associated with a significant tracer uptake when compared to healthy BM [93].

Those studies clearly depict the potential of CXCR4-directed PET imaging as a diagnostic marker in hematologic malignancies. However, its application in the future might be more directed towards patient selection for personalized therapeutic concepts such as CXCR4-directed ERT, rather than to evaluate disease extent or analyze remission upon standard treatments. This is partially due to the highly dynamic CXCR4 expression levels that are particularly volatile after administration of chemotherapy [94]. Therefore, the in vivo determination of CXCR4 levels by means of PET outmatches CXCR4 expression determined by immunochemistry owing to its clinical applicability. In addition, in all the described hematologic malignancies there are either established conventional strategies (cytomorphology, flow cytometry, immunohistochemistry), or molecular markers (serum markers such as immunofixation of monoclonal proteins, or minimal residual disease markers using PCR or sequencing technologies) or an elaborated and well-established imaging modality, e.g., [^18^F]FDG PET, for the evaluation or control of remission available.

However, additional utility for response assessment might be found in CXCR4-directed PET imaging of lymphoma of the central nervous system. It is conceivable that in this lymphoma entity, the CXCR4-directed tracer [^68^Ga]Pentixafor, due to its better contrast characteristics compared to [^18^F]FDG PET in the CNS (unpublished data), may improve the current response criteria provided by the International Primary CNS Lymphoma Collaborative Group [95]. It has recently been shown that response assessment with PET/CT in a patient with extranodal marginal zone lymphoma of the orbital cavities showed comparable results with [^68^Ga]Pentixafor and [^18^F]FDG [96].

#### Imaging solid cancers

CXCR4 overexpression was also found in various solid cancers, including breast, prostate [18–20], lung and colorectal cancer [21–23]. In analogy with hematologic malignancies, high CXCR4 expression in solid tumors is associated with worse prognosis [16, 97]. Yet, first in vivo pilot studies with the CXCR4-directed PET tracer [^68^Ga]Pentixafor revealed a more modest as well as heterogeneous, and in some cases absent detectable receptor expression in solid cancers, a finding contrary to the expected CXCR4 expression profile from in vitro studies [98, 99]. Some solid tumor types, however, did show a pronounced overexpression of CXCR4. Two separate studies found intense, histologically proven CXCR4 expression in most patients with small cell lung cancer [100] as well as non-small cell lung cancer [101]. Furthermore, Bluemel et al. demonstrated feasibility of [^68^Ga]Pentixafor imaging in patients with advanced adrenocortical cancer. In this theranostic approach, about 70% of patients showed sufficient CXCR4 expression to potentially qualify for CXCR4-directed radionuclide therapy [102]. A study by Werner et al. looking at the relationship between tumor grading in neuroendocrine tumors and receptor expression found that an increase in receptor expression correlates with higher tumor grade [103]. Furthermore, in a recently published study, Fang et al. showed increased CXCR4 expression in esophageal malignancies, with most of the signal coming from immune cells (neutrophils and T cells), and not esophageal fibroblasts or endothelial cells [14]. This finding adds to earlier observations, describing that neutrophils contributed to carcinogenesis by secretion of interleukins [104]. A first pilot study also demonstrated feasibility of CXCR4-directed imaging for detection of glioblastoma. However, part of the PET signal may not arise from specific binding of the tracer to CXCR4 but might only be a perfusion effect due to a damaged blood–brain barrier [105].

### CXCR4 imaging in cardiovascular disease

Because of its prominent role in inflammation in general, and in immune cell regulation in particular, various studies have investigated the potential of CXCR4-directed imaging for the detection of hidden infectious foci, or its use for visualizing the extent of conditions accompanied by inflammation, respectively. In one study, [^68^Ga]Pentixafor PET/CT identified altered cerebral CXCR4 expression in a patient who recently suffered a stroke, corresponding well with ischemia-demarcation as assessed by cerebral MRI [106]. Multiple studies, examining CXCR4 expression after acute myocardial infarction, showed, that the PET signal correlated with the extent of infarcted myocardium, as measured by cardiac MRI [107–110]. In addition, CXCR4 expression might reveal the myocardial healing potential, as assessed by follow-up imaging months after acute myocardial infarction [110]. CXCR4-directed PET imaging has also been successfully used to identify atherosclerotic lesions [111–113]. Most likely, the elevated CXCR4 expression in myocardial infarction and atherosclerotic plaques, as measured by [^68^Ga]Pentixafor PET, originates from infiltrating leukocytes to the infarct area and the atherosclerotic lesion, respectively [114, 115]. In a recently published study, Li et al. were able to show anti-inflammatory effects in atherosclerotic lesions of patients that underwent CXCR4-directed ERT [116].

### CXCR4 imaging in infectious diseases

Imaging CXCR4 expression on infiltrating leukocytes might as well be used to track leukocytes that are involved in infectious diseases. In a promising first pilot study, [^68^Ga]Pentixafor PET/CT was able to identify chronic bone infections, with better diagnostic accuracies than anti-granulocyte imaging with ^99m^Tc-besilesomab, or ^99m^Tc-labeled white blood cells, respectively [117]. Results of another study indicate, that CXCR4-directed PET/MRI with [^68^Ga]Pentixafor is able to detect infectious foci by imaging leukocyte infiltration in patients with complicated urinary tract infections after kidney transplantation [118].

## Theranostics

As mentioned before, most tumors have a worsening prognosis with increasing CXCR4 expression [119], although many of the underlying mechanisms and their implications for disease progression are still unknown. For instance, high CXCR4 expression on AML blasts correlates with a poor prognosis [99, 120], and the protective bone marrow environment is considered a major reason for treatment resistance and relapse [121], suggesting potential benefits from CXCR4-directed therapies.

Imaging of CXCR4 expression in oncology has mostly not been of diagnostic nature until now. Instead, it was guided predominantly by theranostic thinking, in pursuit of potential therapeutic remedies for patients with otherwise limited or non-existent treatment alternatives.

## CXCR4-targeted radionuclide therapy

**Fig. 1:**
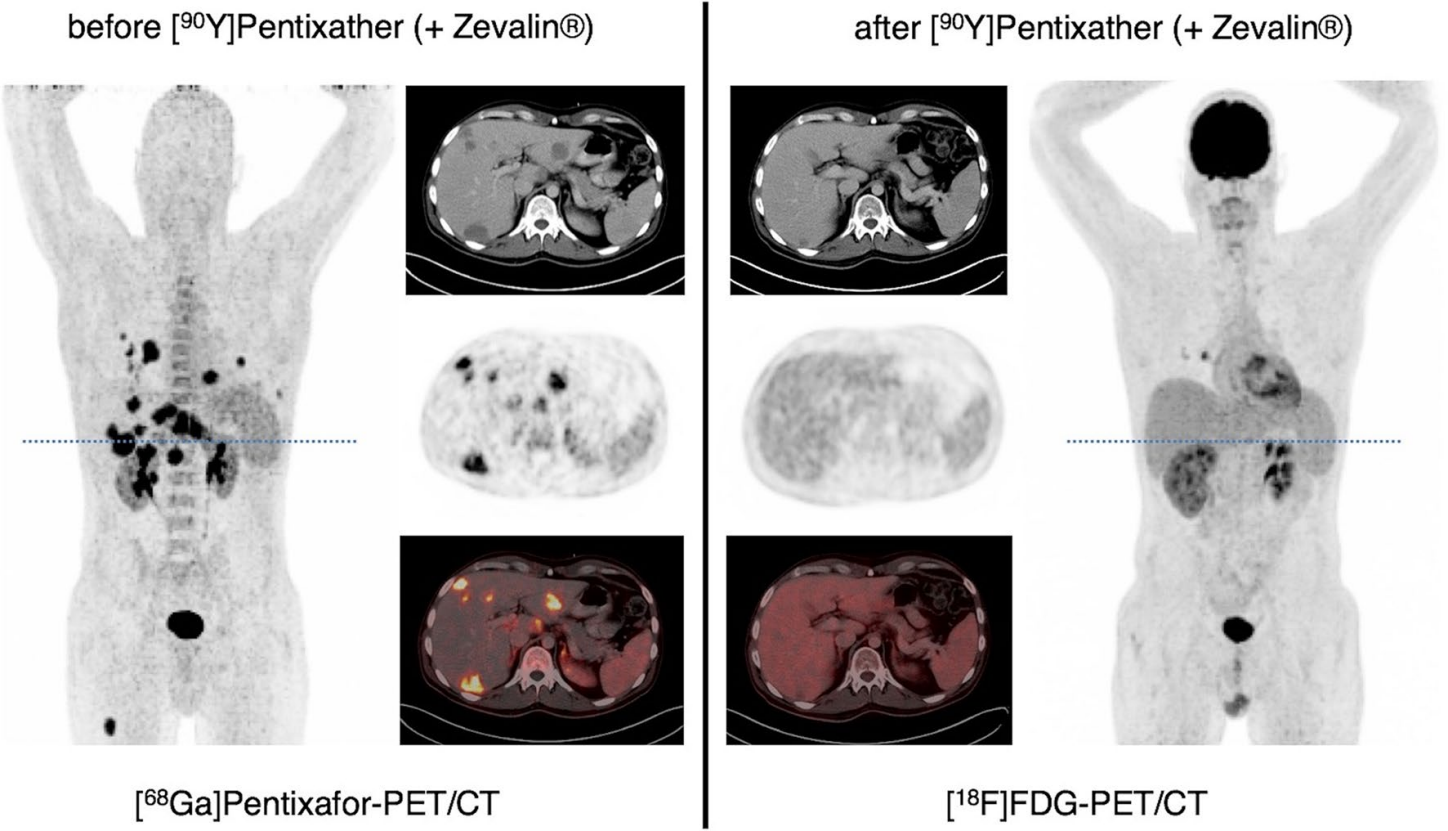
Example of CXCR4-directed endoradiotherapy with [^90^Y]Pentixather (in combination with CD20-directed radioimmunotherapy with [^90^Y]Zevalin^®^) as part of the conditioning regimen prior to allogeneic stem cell transplantation in relapsed/refractory diffuse large B cell lymphoma (DLBCL). Display of maximum intensity projections (outer columns) and transaxial slices (inner columns; CT, upper row, PET, middle row; PET/CT, lower row) of pre-therapeutic CXCR4-directed and post-therapeutic [^18^F]FDG PET/CT. Post-therapeutic imaging was performed 8 weeks after administration of 3.7 GBq of [^90^Y]Pentixather, 1.2 GBq of [^90^Y]Zevalin^®^ as well as conditioning chemotherapy with subsequent repeat stem cell transplantation (SCT) and demonstrated partial response with residual yet vital pulmonary lesions and resolution of all hepatic and nodal DLBCL manifestations. DLBCL had been relapsed from prior first allogeneic SCT and been refractory to all chemotherapeutic regimens

Pentixather, the therapeutic twin of Pentixafor, is a promising CXCR4 ligand that can be labeled with radionuclides for ERT [84]. First studies examined the use of Pentixather, labeled with beta-emitters ^177^Lu or ^90^Y, for ERT of advanced stage MM patients. Although initial response rates were high and adverse effects were limited, overall survival in this very high-risk cohort was not extended [105, 122]. Other pilot investigations showed encouraging results using ERT with [^177^Lu]/[^90^Y]Pentixather in diffuse large B cell lymphoma (DLBCL; see also Fig. 1) [123] and in AML patients, respectively [89]. As of now, there is only one prospective trial for CXCR4-directed ERT in preparation (COLPRIT trial, Eudra-CT 2015-001817-28), that will primarily investigate the tolerable dose and side effects of such ERT in patients with MM or lymphoma.

In all cases until now, ERT was performed in addition to high-dose chemotherapy regimens, followed by subsequent hematopoietic stem cell transplantation. It is noteworthy that in hematologic diseases with the intent to perform allogeneic hematopoietic transplantation, the myeloablation by ERT is considered a desired effect to allow engraftment of the cellular therapeutic. Opposite to that, allogeneic hematopoietic cell transplantation is not an established and suitable approach in other malignancies, and myeloablation induced by binding of the radionuclide to hematopoietic progenitor cells in the bone marrow is certainly of major concern. ERT without stem cell rescue might be technically feasible for tumors with pronounced receptor overexpression as witnessed in adrenocortical cancer or small cell lung cancer but requires further development and prospective investigations.

## Outlook

CXCR4-directed ERT, and particularly—imaging— is rapidly gaining popularity at a few academic centers. The (pre-)clinically observed dynamics in CXCR4 expression (e.g., chemotherapy-induced) present new opportunities to potentially modulate CXCR4 expression and function. By prior receptor upregulation, imaging might benefit from higher sensitivities, and anti-cancer therapies might find more targets on the cell surface for a stronger effect. Similarly, downregulation of CXCR4 might have synergistic effects with conventional therapies. Furthermore, labeling a CXCR4 ligand with an alpha-emitter for ERT might present a breakthrough in hematologic malignancies, as the higher energy transfer would lead to more effective destruction of cancer and cancer-supporting cells.

## Summary

CXCR4 and its natural ligand, the chemokine CXCL12, play important physiologic roles in embryonic development, hematopoiesis and immunity. But the CXCR4-CXCL12 axis is also deeply associated with disease and is particularly involved in tumor growth and metastasis. A multitude of different malignancies overexpress CXCR4 on their respective cell surface, which predominantly is associated with a worse prognosis. Different therapeutics targeting CXCR4 or its ligand CXCL12 have been developed. For instance, Plerixafor, the first FDA-approved CXCR4 inhibitor, mobilizes stem-/progenitor cells from the bone marrow into the circulation. Subsequently, various CXCR4-directed imaging tracers were developed, with the positron-emitting PET tracer [^68^Ga]Pentixafor being the most frequently used today. CXCR4 imaging with [^68^Ga]Pentixafor has successfully been performed in several different malignancies, as well as in cardiovascular disease and infections. Its therapeutic twin, Pentixather, labeled with the beta-emitters ^177^Lu or ^90^Y, has already been used for ERT in various hematologic malignancies. Research in the field of CXCR4-directed imaging and radionuclide therapy is highly active, and new developments over the full spectrum of translational medicine are anticipated in the coming years.
